# Supplementary material for: Complexome profiling of the Chlamydomonas psb28 mutant reveals TEF5 as an early PSII assembly factor
Source: Plant Cell. 2025 Mar 17;37(6):koaf055. doi: 10.1093/plcell/koaf055 (PMC12164590; doi:10.1093/plcell/koaf055)
Supplement: koaf055_Supplementary_Data [file koaf055_supplementary_data.zip › psb28_interactive.zip!/plots/intensity/qualityPlots/peptides/SPP1B_Cre06.g267700.t1.2.html]

### Cre06.g267700.t1.2

Trivial name: SPP1B  
SeqCoverage [median]: 4.90  
Mass: 76.53 kDa  
  
 

| Sequence | MissedCleavage | Mass [Da] | wtIntensity | muIntensity | Score |
| --- | --- | --- | --- | --- | --- |
| GDTQQVELPK | 0 | 1113.57 | 256.29 | 0.00 | 65.22 |
| SAANTFR | 0 | 765.38 | 0.00 | 400.24 | 77.06 |
|  |  |  | 256.3 | 400.2 |  |
